# Supplementary material for: Harnessing the regenerative potential of interleukin11 to enhance heart repair
Source: Nat Commun. 2024 Nov 8;15:9666. doi: 10.1038/s41467-024-54060-0 (PMC11549343; doi:10.1038/s41467-024-54060-0)
Supplement: Supplementary file 2 — Description of Additional Supplementary Files [file 41467_2024_54060_MOESM2_ESM.pdf]

Description of Additional Supplementary Files

Supplementary Data 1: This file contains bulk RNA-seq analysis to determine differentially expressed gene list of 7 dpt *il11a*OE.

Supplementary Data 2: This file contains bulk RNA-seq analysis to determine differentially expressed gene list of 7 mpt *il11a*OE.
