# Supplementary material for: Harnessing the regenerative potential of interleukin11 to enhance heart repair
Source: Nat Commun. 2024 Nov 8;15:9666. doi: 10.1038/s41467-024-54060-0 (PMC11549343; doi:10.1038/s41467-024-54060-0)
Supplement: Supplementary file 5 — Reporting Summary [file 41467_2024_54060_MOESM5_ESM.pdf]

nature portfolio

Corresponding author(s): Junsu Kang

Last updated by author(s): Oct 2, 2024

Reporting Summary

Nature Portfolio wishes to improve the reproducibility of the work that we publish. This form provides structure for consistency and transparency in reporting. For further information on Nature Portfolio policies, see our Editorial Policies and the Editorial Policy Checklist.

Statistics

For all statistical analyses, confirm that the following items are present in the figure legend, table legend, main text, or Methods section.

n/a	Confirmed	
☐	☒	The exact sample size (*n*) for each experimental group/condition, given as a discrete number and unit of measurement
☐	☒	A statement on whether measurements were taken from distinct samples or whether the same sample was measured repeatedly
☐	☒	The statistical test(s) used AND whether they are one- or two-sided<br>*Only common tests should be described solely by name; describe more complex techniques in the Methods section.*
☒	☐	A description of all covariates tested
☒	☐	A description of any assumptions or corrections, such as tests of normality and adjustment for multiple comparisons
☐	☒	A full description of the statistical parameters including central tendency (e.g. means) or other basic estimates (e.g. regression coefficient) AND variation (e.g. standard deviation) or associated estimates of uncertainty (e.g. confidence intervals)
☐	☒	For null hypothesis testing, the test statistic (e.g. *F*, *t*, *r*) with confidence intervals, effect sizes, degrees of freedom and *P* value noted<br>*Give P values as exact values whenever suitable.*
☒	☐	For Bayesian analysis, information on the choice of priors and Markov chain Monte Carlo settings
☒	☐	For hierarchical and complex designs, identification of the appropriate level for tests and full reporting of outcomes
☒	☐	Estimates of effect sizes (e.g. Cohen's *d*, Pearson's *r*), indicating how they were calculated

Our web collection on statistics for biologists contains articles on many of the points above.

Software and code

Policy information about availability of computer code

Data collection	Bright-field images and AFOG staining images were captured using BZ-X810 fluorescence microscope (Keyence).<br>Images of cardiac tissue sections were acquired using an Eclipse Ti-U inverted compound microscope (Nikon) or BZ-X810 fluorescence microscope (Keyence).<br>For RNA sequencing, generation of mRNA libraries and sequencing were performed at the Biotechnology center at UW-Madison using an Illumina NovaSeq with 150 bp paired-end runs.<br>Reanalysis of single cell RNA sequencing was performed with count files from GSE159032 and GSE158919 .
Data analysis	Fluorescence images were processed using either NIS-Elements (Nikon), ZEN (Zeiss, v3.3), BZ-X800 analyzer (Keyence, 1.1.1.8) or FIJI/ImageJ software (2.0.0-rc-66/1.52n). Further process was performed in Adobe Photoshop (2023 or 2024). The quantification was performed using FIJI/ImageJ software.<br>For RNA seq, computational analysis was performed in Linux (Ubuntu20.4) and R (4.2 ~ 4.3.1).<br>Adaptor sequences were trimmed by Cutadapt. Sequences were aligned to the zebrafish genome (GRCz11) using HISAT2 (v2.2.1).<br>Differentially regulated transcripts were identified using Featurecount and Deseq2 (v1.40.2). GO-term and GSEA analyses were done by the enrichGo and gseGO functions of ClusterProfiler (v4.8.3).<br>For single cell RNA seq, and reanalyzed with Seurat package (v5.0.3).

For manuscripts utilizing custom algorithms or software that are central to the research but not yet described in published literature, software must be made available to editors and reviewers. We strongly encourage code deposition in a community repository (e.g. GitHub). See the Nature Portfolio guidelines for submitting code & software for further information.

Data

Policy information about availability of data

All manuscripts must include a data availability statement. This statement should provide the following information, where applicable:

- Accession codes, unique identifiers, or web links for publicly available datasets
- A description of any restrictions on data availability
- For clinical datasets or third party data, please ensure that the statement adheres to our policy

Data associated with this study are presented in the paper or in the Supplementary Materials. RNA-sequencing data are deposited in GEO under the accession code GSE233833. Source data are provided.

Research involving human participants, their data, or biological material

Policy information about studies with human participants or human data. See also policy information about sex, gender (identity/presentation), and sexual orientation and race, ethnicity and racism.

Reporting on sex and gender	N/A
Reporting on race, ethnicity, or other socially relevant groupings	N/A
Population characteristics	N/A
Recruitment	N/A
Ethics oversight	N/A

Note that full information on the approval of the study protocol must also be provided in the manuscript.

Field-specific reporting

Please select the one below that is the best fit for your research. If you are not sure, read the appropriate sections before making your selection.

[x] Life sciences [ ] Behavioural & social sciences [ ] Ecological, evolutionary & environmental sciences

For a reference copy of the document with all sections, see nature.com/documents/nr-reporting-summary-flat.pdf

Life sciences study design

All studies must disclose on these points even when the disclosure is negative.

Sample size	No methods were used to predetermine the sample sizes. Sample sizes were chosen based on previous publications (PMIDs: 35179181, 25938716, and 35652354) and were clearly indicated in the figure legends.
Data exclusions	No data was excluded.
Replication	All animal experiments were performed on at least three biological replicates per group and confirmed with successful replication.
Randomization	Clutchmates, or hearts collected from clutchmates, were randomized into different group of each treatment.
Blinding	The investigators were blinded to group allocation during data analysis and quantification was performed unbiasedly.

Reporting for specific materials, systems and methods

We require information from authors about some types of materials, experimental systems and methods used in many studies. Here, indicate whether each material, system or method listed is relevant to your study. If you are not sure if a list item applies to your research, read the appropriate section before selecting a response.

Materials & experimental systems

n/a	Involved in the study	
☐	☒	Antibodies
☒	☐	Eukaryotic cell lines
☒	☐	Palaeontology and archaeology
☐	☒	Animals and other organisms
☒	☐	Clinical data
☒	☐	Dual use research of concern
☒	☐	Plants

Methods

n/a	Involved in the study	
☒	☐	ChIP-seq
☒	☐	Flow cytometry
☒	☐	MRI-based neuroimaging

Antibodies

Antibodies used

The primary antibodies: anti-myosin heavy chain (mouse, F59; Developmental Studies Hybridoma Bank; 1:50 or mouse, MF20; Developmental Studies Hybridoma Bank; 1:50), anti-EGFP (rabbit, A11122; Life Technologies; 1:200), anti-EGFP (chicken, GFP-1020; Aves Labs; 1:2000), anti-Ds-Red (rabbit, 632496; Clontech; 1:500), anti-mCherry (chicken, MC87977980; Aves Labs; 1:500), anti-Raldh2 (rabbit, GTX124302; Genetex; 1:200), anti-PCNA (mouse, P8825; Sigma; 1:200), anti-α-actinin (mouse, A7811; Sigma, 1:200), anti-ACTA2 (rabbit, GTX124505; GeneTex; 1:200), anti-pERK (rabbit, 9101; Cell Signaling Technology; 1:250). anti-Mef2 (rabbit; 1:200), Anti-phospho-Histone H3 (rabbit, 9701; Cell Signaling Technology, 1:100), Anti-phospho STAT3 (rabbit, 9131S; Cell Signaling Technology, 1:100), anti-Periostin (rabbit, 19899-1-AP, Proteintech, 1:200)
The secondary antibodies: Alexa Fluor 488 (mouse, rabbit, and chicken; A11029, A11034, and A11039; Life Technologies; 1:500), Alexa Fluor 594 (mouse and rabbit; A11032 and A11037; Life Technologies; 1:500)

Validation

All primary antibodies, except Anti-Mef2, commercially available and are validated by suppliers as follows.
F59: https://dshb.biology.uiowa.edu/F59
MF20: https://dshb.biology.uiowa.edu/MF-20
EGFP: https://www.thermofisher.com/antibody/product/GFP-Antibody-Polyclonal/A-11122 (rabbit)
https://www.aveslabs.com/products/anti-green-fluorescent-protein-antibody-gfp(chicken)
Ds-Red: https://www.takarabio.com/products/antibodies-and-elisa/fluorescent-protein-antibodies/red-fluorescent-protein-antibodies
mCherry: https://www.aveslabs.com/products/anti-mcherry-antibody-mcherry
Raldh2: https://www.genetex.com/Product/Detail/Aldh1a2-antibody/GTX124302
PCNA: https://www.sigmaaldrich.com/US/en/product/sigma/p8825
α-actinin: https://www.sigmaaldrich.com/US/en/product/sigma/a7811
ACTA2: https://www.genetex.com/Product/Detail/Acta2-antibody/GTX124505
pERK: https://www.cellsignal.com/products/primary-antibodies/phospho-p44-42-mapk-erk1-2-thr202-tyr204-antibody/9101
pH3: https://www.cellsignal.com/products/primary-antibodies/phospho-histone-h3-ser10-antibody/9701
pSTAT3: https://www.cellsignal.com/products/primary-antibodies/phospho-stat3-tyr705-antibody/9131
Periostin: https://www.ptglab.com/products/POSTN-Antibody-19899-1-AP.htm
Anti-Mef2 were validated in the previous publication: https://doi.org/10.1038/s41467-022-35433-9

Animals and other research organisms

Policy information about studies involving animals; ARRIVE guidelines recommended for reporting animal research, and Sex and Gender in Research

Laboratory animals

Wild-type or transgenic zebrafish of the outbred Ekkwill (EK) or AB strains ranging up to 18 months of age were used for all zebrafish experiments.
Figure 1a and b: 4 - 6 month
Figure 2b and d: 4 - 6 month
Figure 2c and e: 4 - 6 month
Figure 2f and g: 4 - 6 month
Figure 2h and i: 4 - 6 month
Figure 2k and l: 4 - 6 month
Figure 3a and b: 4 - 6 month
Figure 3c and d: 4 - 6 month
Figure 3h and i: 4 - 6 month
Figure 4a and b: 12 - 18 month
Figure 4d and e: 12 - 18 month
Figure 4f and g: 4 - 6 month
Figure 4h and i: 4 - 6 month
Figure 5a and b: 4 - 6 month
Figure 5c and d: 4 - 6 month
Figure 5g and h: 4 - 6 month
Figure 5i and j: 4 - 6 month
Sup. figure 1c: 5 days post-fertilization

	Sup. figure 1d and e: 4 - 6 month<br>Sup. figure 2a and b: 4 - 6 month<br>Sup. figure 3a and b: 4 - 6 month<br>Sup. figure 4: 4 - 6 month<br>Sup. figure 5: 12 month<br>Sup. figure 7c and d: 4 - 6 month<br>Sup. figure 8a and b: 4 - 6 month<br>Sup. figure 8c and d: 4 - 6 month<br>Sup. figure 9b and c: 4 - 6 month<br>Sup. figure 10a and b: 4 - 6 month<br>Sup. figure 10c and d: 4 - 6 month<br>Sup. figure 10e and f: 4 - 6 month<br>Sup. figure 11a and b: 4 - 6 month
Wild animals	No wild animal used
Reporting on sex	Animals of both sexes were used for experiments.
Field-collected samples	No field-collected samples
Ethics oversight	Work with zebrafish was approved and performed in accordance with University of Wisconsin-Madison guidelines.

Note that full information on the approval of the study protocol must also be provided in the manuscript.

Plants

Seed stocks	N/A
Novel plant genotypes	N/A
Authentication	N/A
